# Success rates of MRSA decolonization and factors associated with failure

**DOI:** 10.1186/s13756-022-01177-w

**Published:** 2022-11-22

**Authors:** Wing-Kee Yiek, Mirjam Tromp, Riet Strik-Albers, Koen van Aerde, Nannet van der Geest-Blankert, Heiman F. L. Wertheim, Corianne Meijer, Alma Tostmann, Chantal P. Bleeker-Rovers

**Affiliations:** 1 Department of Internal Medicine, Radboudumc Center for Infectious Diseases, Radboud University Medical Center, P O Box 9101, 6500 HB Nijmegen, The Netherlands; 2 Department of Paediatric Infectious Disease and Immunology, Radboud University Medical Center, Radboudumc Institute for Molecular Life Sciences, Radboudumc Amalia Children’s Hospital, Nijmegen, The Netherlands; 3 Department of Occupational Health, Radboud University Medical Center, Nijmegen, The Netherlands; 4 Department of Medical Microbiology, Radboudumc Center for Infectious Diseases, Radboud University Medical Center, Nijmegen, The Netherlands

**Keywords:** Colonization, Decolonization, MRSA, Treatment success

## Abstract

**Background:**

We evaluated the success rate of MRSA decolonization directly after treatment and after one year in patients who were treated at the outpatient MRSA clinic of a large university medical centre to identify potential contributing factors to treatment success and failure.

**Methods:**

Data from November 1, 2013 to August 1, 2020 were used. Only patients who had undergone complete MRSA decolonization were included. Risk factors for MRSA treatment failure were identified using a multivariable logistic regression model.

**Results:**

In total, 127 MRSA carriers were included: 7 had uncomplicated carriage, 91 had complicated carriage, and 29 patients had complicated carriage in combination with an infection. In complicated carriers and complicated carriers with an infection final treatment was successful in 75.0%. Risk factors for initial treatment failure included having one or more comorbidities and not testing the household members. Risk factors for final treatment failure were living in a refugee centre, being of younger age (0–17 years), and having one or more comorbidities.

**Conclusions:**

The results of this study indicate that patients with a refugee status and children treated at the paediatric clinic have a higher risk of MRSA decolonisation treatment failure. For this reason, it might be useful to revise decolonization strategies for these subgroups and to refer these patients to specialized outpatient clinics in order to achieve higher treatment success rates.

## Introduction

Methicillin-resistant *Staphylococcus aureus* (MRSA) is a human pathogen and an important cause of a wide range of infections. MRSA has become endemic in health care institutions worldwide. Carriage of MRSA is associated with a higher risk of infection than carriage of methicillin-susceptible *Staphylococcus aureus*. [1, 2]. In the Netherlands, the MRSA prevalence in 2018 was only 1.2% [3]. This low prevalence can be explained by the national search and destroy (S&D) policy in combination with careful and restrictive antibiotic use. The S&D policy focuses on isolation of MRSA carriers and of patients with an increased risk of MRSA carriage, outbreak management, and follow-up of MRSA carriers. The goal of this policy is successful eradication of MRSA carriage [4–6].

MRSA eradication treatment serves two purposes: the prevention of infection and the prevention of further transmission. MRSA treatment success rates directly after the decolonization attempt have been reported in one previous study published in 2011, showing a success rate of 56% among complicated MRSA carriers. This study recommended to testing household members of MRSA carriers and treating them simultaneously as the index case if they are positive [2]. This was included in the most recent national Dutch protocol in 2012 [7]. The success rates after implementation of these updated guidelines have not been evaluated yet. The success rate after one year of follow-up, when the absence of MRSA-carriage is considered to be definite, has not been reported before. Evaluating therapy success rates and determinants for therapy failure can contribute to further improvement of MRSA decolonization strategies. Additionally, new potential risk factors may have arisen in the past years. To our knowledge, this is the first study on MRSA decolonization that takes refugee status into account as potential risk factor.

## Objectives

The aim of this study was to evaluate the success rate of MRSA decolonization directly after treatment as well as after one year among patients who were treated at the outpatient MRSA clinic of a large university medical centre in order to identify potential contributing factors for success that could be used to further improve treatment.

### Patients and methods

#### Study design and study population

A retrospective cohort study was conducted among patients who had started and finished MRSA decolonization treatment for MRSA carriage with follow-up cultures taken between November 1, 2013 and August 1, 2020 in the Radboud university medical centre (Radboudumc). Only patients who had undergone complete MRSA decolonization were included. Patients who were treated solely for MRSA infection or load reduction prior to a surgical procedure were not included. Patients were also excluded if their medical records and/or microbiological culture results were unavailable. Those who did not complete the first three follow-up culture rounds were also excluded.

#### MRSA treatment protocol

The SWAB (Dutch Working Party on Antibiotic Policy) protocol for treating MRSA carriers is used as standard MRSA treatment protocol in Dutch hospitals [7]. Table 1 shows a short summary of this treatment protocol and illustrates the differences and similarities between the treatment of uncomplicated and complicated MRSA carriage.

**Table 1:**
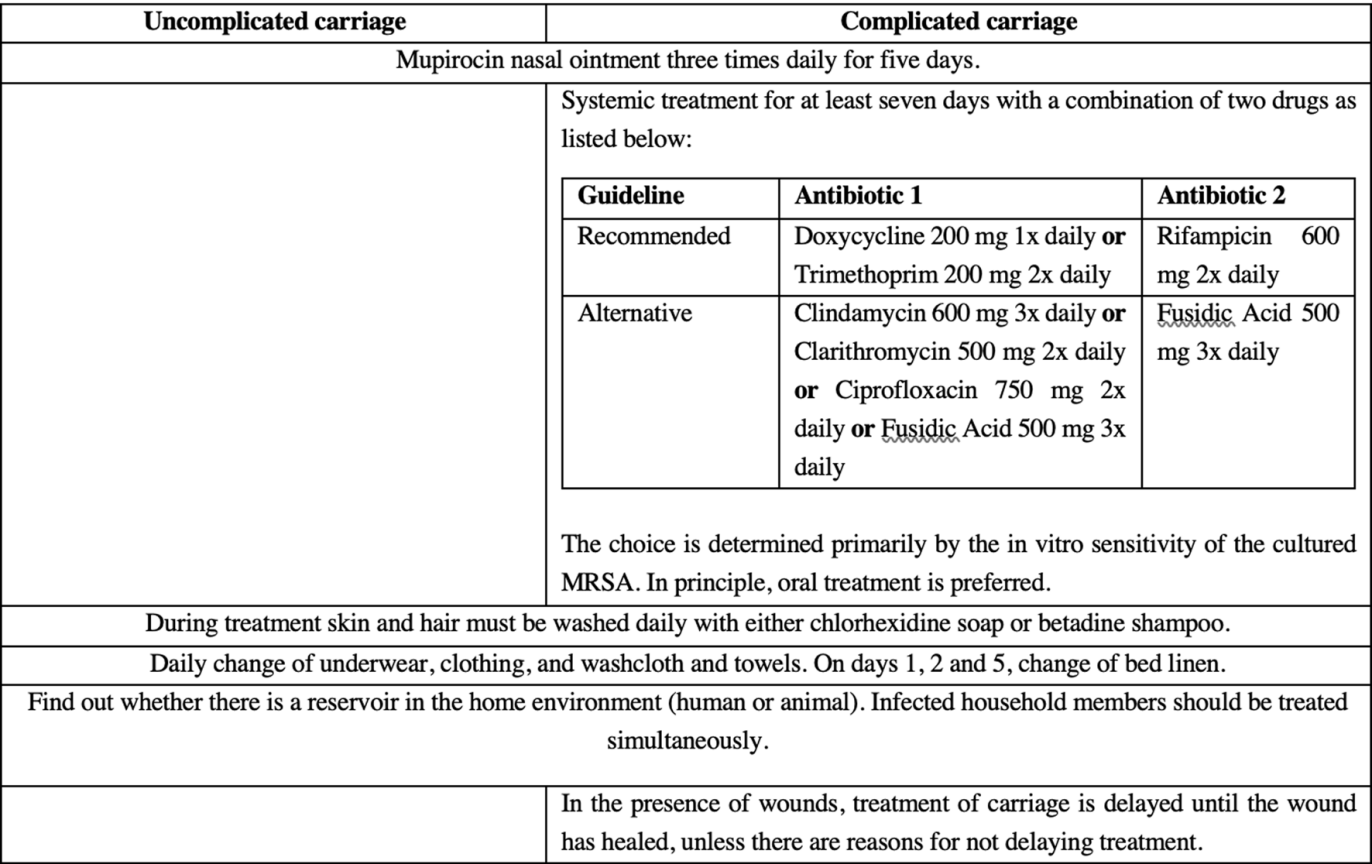
SWAB recommendations regarding treatment of MRSA carriage [7]

#### Definitions and outcomes

Uncomplicated and complicated MRSA were defined according to Dutch MRSA treatment guidelines [7, 8].

In uncomplicated MRSA carriage:

- The MRSA is exclusively localized in the nose,
- There is no active infection and there are no skin lesions,
- The MRSA is sensitive in vitro to the antibiotics to be prescribed,
- And there is no foreign material that forms a connection between the internal and the external patient environment.

For complicated MRSA carriage at least one of the following criteria are met:

- Located in throat, perineum, or skin lesions, independent of nasal carriage
- There are active skin lesions
- There is foreign material that forms a connection between the internal and the external environment
- MRSA is in vitro resistant to mupirocin
- Previous treatments according to the recommendations for uncomplicated carriage have failed.

Patients who had an active infection in addition to their MRSA carriage were categorized as ‘Infection and complicated carriage’. When looking at the treatment success rates, this group was merged with the complicated carriers.

Treatment outcome was assessed at two timepoints and for each patient we determined ‘initial treatment success’ and ‘final treatment success’. Treatment outcome was either ‘successful’ or ‘failed’.

**Fig. 1:**
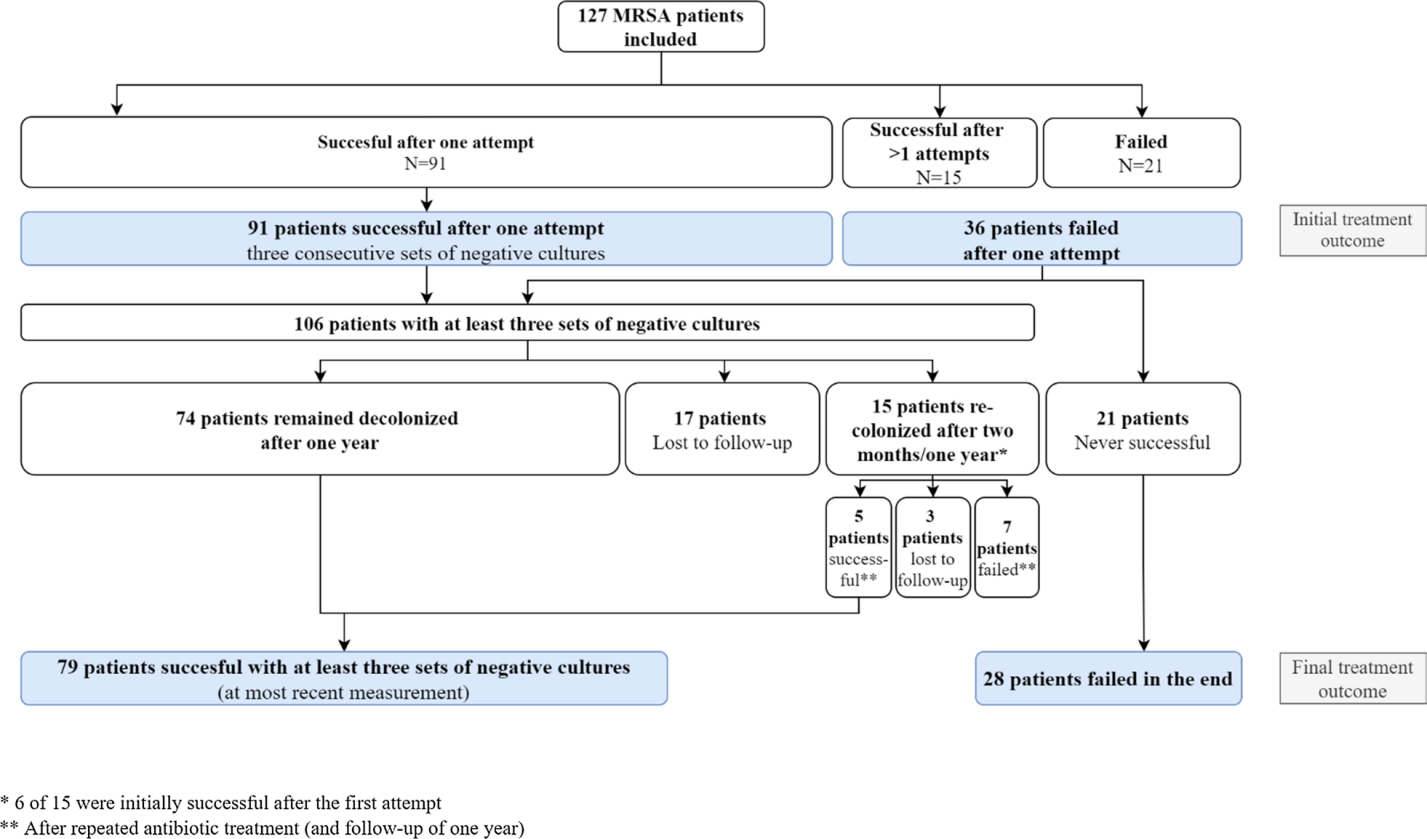
Treatment outcomes of MRSA decolonization treatment

Initial treatment success was defined as three consecutive negative culture sets obtained at least 48 h after completion of the first treatment and distributed over a period of at least seven days. When a patient also had negative MRSA cultures after two months and one year, they were defined as having final treatment success. Patients in whom decolonization treatment was not attempted after one or multiple failed treatments and remained MRSA carriers were defined as having treatment failure. Patients who had ‘initial treatment success’ but who later had positive cultures again were considered ‘recurrent MRSA carriers’. Patients who missed the two-month and/or the one-year cultures were included in the study but were marked as ‘lost to follow-up’ for determination of final treatment outcome, as it remains unknown if there was a recurrence of MRSA in these patients. Therefore, the patients who did not complete the full follow-up of one year were excluded in the analysis of final treatment outcome. Figure 1 shows an overview of the patient treatment outcomes of MRSA decolonization treatment.

#### Data collection

Data were collected from the electronic patient system (EPIC). Information on MLST type was collected from the Medical Microbiology Laboratory database and the National MRSA Surveillance database Type-Ned from the Dutch Institution of Public Health and the Environment. A list of possible risk factors and determinants for treatment success were retrieved from literature and expert opinion. Data were entered in a secure research data environment in a database built in Castor.

The source of infection was divided into five categories: unknown, hospital-acquired, livestock-associated, community-acquired, and living in a refugee centre. An unknown source was specified as a patient without any known risk factor. Hospital-acquired MRSA was defined as any hospital admission in the Netherlands in the past year where the patient came into contact with an MRSA positive person and patients who were admitted or treated in a hospital outside the Netherlands. MRSA from patients who were involved with livestock were categorized as livestock-associated MRSA. MRSA from patients who had contact with confirmed MRSA positive persons outside the hospital were categorized as community-acquired MRSA. Refugee status was categorized based on living in a refugee centre or otherwise. In cases where patients could be placed in more than one category, a decision was made based on the most likely origin. Household members of an index patient were defined as a person staying in the same house during the day and night and having at least one shared facility [7].

#### Statistical analysis

For the data analysis, Statistical Package for the Social Sciences (SPSS) v25 and RStudio v1.2.5033 were used. Patients were divided based on their initial and final treatment outcomes and initial and final MRSA decolonization treatment success rates were calculated. Univariable logistic regression analysis was done to identify determinants for initial and final treatment failure for categorical variables. The Fisher’s exact test was used for dichotomous variables. *P* values were not calculated for variables with sample sizes smaller than five. Age was divided into three age groups: 0–17 years, 18 to 64 years, and 65 years and older. Patients under 18 years were treated at the paediatric MRSA outpatient clinic. Household size was categorized in three groups: 1–2, 3–4, and 5 or more household members, and was categorized as ‘unknown’ if this information was unavailable.

Multivariable logistic regression analysis was then performed to assess the associations between determinants of interest and both initial treatment outcome (i) and final treatment outcome (ii). All variables with a p value of < 0.1 in the univariable analysis were included in the multivariable analysis. Before entering these variables in the multivariable analysis, all potential variables were checked for multicollinearity by using the Variance Inflation Factor (VIF). Variables with a VIF larger than 4.0 were removed from the final model. Significance was defined as a p value of < 0.05. The enter method was chosen for the multivariable analysis. The results were presented by using adjusted odds ratios (OR) with their respective 95% confidence intervals (CI).

## Results

### Baseline characteristics

We identified 127 patients who started and finished MRSA decolonization therapy in the study period. Table 2 shows the patient characteristics of the study population and Table 4 shows the patient characteristics of adults and children separately. Of all the patients, 30.7% had one or more of the following comorbidities: chronic lung disease, skin disease, renal disease that required a solid organ transplant (SOT) or dialysis, or the need of indwelling devices. Indwelling devices were specified as all foreign material that forms a connection between the internal and external environment. There were 21 patients (16.5%) living in a refugee centre, including 15 children. Exposure to livestock was present in 16.5% of the patients. Other MRSA sources were hospital-acquired (23.6%), community-acquired (26.0%), and unknown (17.3%).

**Table 2:** Patient characteristics of MRSA carriers and determinants associated with initial treatment failure

	Total (*n* = 127)	Initial treatment success (*n* = 90)	Initial treatment failure (*n* = 37)	Crude Odds Ratio [95% CI]	*P*-value uni-variable	Adjusted Odds ratio [95% CI]	*P*-value multi-variable
Age, median	33.0	40	17				
*Age groups, n (%)*							
0–17	35	15 (42.9)	20 (57.1)	5.62 [2.31–13.65]	< 0.001	5.85 [1.80–18.99]	< 0.01
18–64	73	59 (80.8)	14 (19.2)	Reference		Reference	
65 +	19	16 (84.2)	3 (15.8)	0.84 [0.20–3.10]	0.74	0.96 [0.19–4.92]	0.96
*Gender, n (%)*							
Male Female	66 61	49 (74.2) 41 (67.2)	17 (25.8) 20 (32.8)	Reference 0.71 [0.31–1.64]	0.44		
*Healthcare worker (HCW), n (%)*							
No Yes	112 15	78 (69.6) 12 (80.0)	33 (30.4) 3 (20.0)	Reference 0.58 [0.10–2.32]	0.55		
*Contact with livestock, n (%)*							
No Yes/in the past	105 22	71 (67.6) 19 (86.4)	4 (32.4) 3 (13.6)	Reference 0.33 [0.06–1.24]	0.12		
*Pets*							
No Yes	92 35	65 (70.7) 26 (74.3)	27 (29.3) 9 (25.7)	Reference 0.96 [0.36–2.43]	1		
*MRSA source, n (%)*							
Unknown ^a^	22	18 (81.8)	4 (18.2)	Reference			
Hospital-acquired	30	22 (73.3)	8 (26.7)	1.64 [0.42–6.33]	0.48	1.83 [0.34–9.76]	0.48
Livestock-associated	21	17 (81.0)	4 (19.0)	1.06 [0.31–4.92]	0.94	2.02 [0.34–12.01]	0.44
Community-acquired	33	25 (75.8)	8 (24.8)	1.44 [0.38–5.52]	0.60	1.98 [0.40–9.84]	0.40
Living in refugee centre	21	8 (38.1)	13 (61.9)	7.31 [1.81–29.54]	< 0.01	8.43 [1.14–62.56]	0.04
*Comorbidity, n (%) *^*b*^							
No Yes	88 39	68 (77.3) 22 (56.4)	20 (22.7) 17 (43.6)	Reference 2.93 [1.13–7.69]	0.02	Reference 3.37 [1.24–9.15]	0.02
Chronic pulmonary disease	10	6 (60.0)	4 (40.0)	1.69 [0.33–7.66]	0.48		
Skin disease	12	7 (58.3)	5 (41.7)	1.84 [0.43–7.32]	0.33		
Renal disease ^c^	7	3 (42.9)	4 (57.1)	3.48 [0.56–25.01]	0.19		
Devices ^d^	18	9 (50.0)	9 (50.0)	2.87 [0.91–9.08]	0.05		
*AB use in the past 3 months, n (%) *^*e*^							
No	99	71 (71.7)	28 (28.3)	Reference	0.81		
Yes	28	19 (67.9)	9 (32.1)	1.20 [0.42–3.20]			
*Hospital admission in the Netherlands in the past year, n (%) *^*e*^							
No	101	73 (72.3)	28 (27.7)	Reference	0.48		
Yes	26	17 (65.4)	9 (34.6)	1.38 [0.48–3.74]			
Skin lesions, *n* (%)							
No	84	58 (69.0)	26 (31.0)	Reference	0.68		
Yes	43	32 (74.4)	11 (25.6)	0.77 [0.30–1.87]			
*Travel abroad in the past year, n (%) *^*e,*^^f^							
No	86	64 (74.4)	22 (25.6)	Reference	0.22		
Yes	41	26 (65.0)	15 (36.6)	1.67 [0.69–4.00]			
*Type of MRSA carriage, n (%)*							
Uncomplicated carriage	7	5 (71.4)	2 (5.6)	Reference			
Complicated carriage	91	64 (70.3)	27 (29.7)	1.05 [0.19–5.78]	0.95		
Infection & Complicated carriage	29	21 (72.4)	8 (27.6)	0.95 [0.15–5.94]	0.96		
*Location of MRSA carriage, n (%)*							
Nasal carriage	70	52 (74.3)	18 (25.7)	0.69 [0.30–1.60]	0.43		
Non-nasal carriage	57	38 (67.9)	19 (32.1)	1.44 [0.62–3.35]	0.43		
Throat carriage	83	56 (67.5)	27 (32.5)	1.63 [0.66–4.27]	0.31		
Perineal carriage	52	41 (78.8)	11 (21.2)	0.51 [0.20–1.22]	0.12		
Other sites	34	26 (76.5)	8 (23.5)	0.68 [0.24–1.79]	0.51		
*MLST*							
ST398	31	25 (80.6)	6 (19.4)	Reference			
ST1	5	4 (80.0)	1 (20.0)	1.04 [0.10–11.09]	0.97		
ST8 and ST281 ^g^	9	4 (44.4)	5 (55.6)	5.21 [1.06–25.50]	0.04		
ST22	15	7 (46.7)	8 (53.3)	4.76 [1.23–18.37]	0.02		
ST30	13	10 (76.9)	3 (23.1)	1.25 [0.26–6.00]	0.78		
ST1933	8	5 (62.5)	3 (37.5)	2.50 [0.46–13.49]	0.29		
Other or unknown	46	35 (76.1)	11 (23.9)	1.31 [0.43–4.01]	0.64		
*PVL positivity, n (%)*							
No Yes	104 23	74 (71.2) 16 (69.6)	30 (28.8) 7 (28.8)	Reference 1.07 [0.34–3.12]	1		
*Side-effects of treatment*							
No Yes	104 23	73 (70.2) 17 (73.9)	31 (29.8) 6 (26.1)	Reference 0.83 [0.245–2.48]	0.80		
Household tested							
Yes No ^h^	117 10	86 (73.5) 4 (40.0)	30 (26.5) 6 (60.0)	Reference 4.11 [0.91–21.16]	0.06	Reference 7.42 [1.36–40.41]	0.02
*Size household*							
1–2 3–5 > 5 and unknown ^i^	44 57 26	36 (81.8) 40 (70.2) 14 (53.8)	8 (18.2) 17 (29.8) 12 (46.2)	Reference 1.91 [0.74–4.96] 3.86 [1.30–11.44]	0.18 0.01	0.85 [0.21–3.43] 0.53 [0.09–3.03]	0.82 0.47

^a^ Known risk factors are: previous MRSA carriage, contact with livestock, contact with MRSA positive person, hospital admission outside the Netherlands

^b^ The comorbidities mentioned below were merged together to get a larger sample size

^c^ Solid organ transplant (SOT) or dialysis

^d^ Tubing, catheters, probes, cannulas etc.

^e^ Measured counting from first visit infectious diseases consult

^f^ Travelling either to Asia, Africa, Central America or South America

^g^ Strains associated with refugees[15]

^h^ When one or more household members were not tested for MRSA, the index patient was categorized as ‘No’

^i^ Unknown cases included refugees who fled alone, but had shared facilities at the refugee centre

### Determinants of initial treatment failure

The initial treatment outcome was successful in 70.9% (90/127) of all patients. In complicated carriers the success rate was 70.8% (85/120). In uncomplicated carriers this was 71.4% (5/7). In the univariable analysis (Table 2), the following variables were independently associated with initial treatment failure: age 0–17 years old, having one or more registered comorbidities, living in a refugee centre, certain MLST types, not testing household members, and a household size of 5 or more members. These variables were used for the multivariable analysis, with an exception of MLST due to high multicollinearity with the MRSA source, as certain sequences are predominantly found in specific sources. Age 0–17 (OR_a_ 5.9, 95% CI [1.8–19.0]); comorbidities (OR_a_ 3.4, 95% CI [1.2–9.2]) and not testing household members (OR_a_ 7.4, 95% CI [1.4–40.4]) were all associated with failure of the first decolonization treatment.

### Determinants for final treatment failure

Of the total group of 127 patients, 20 patients were excluded in this analysis due to loss to follow-up at two months (*n* = 14) or at one year (*n* = 6). The final treatment outcome was successful in 73.8% (79/107) of all patients. In complicated carriers, treatment was successful in 75.0% (75/100) and in uncomplicated carriers this was 57.1% (4/7). The univariable analysis in Table 3 shows the same determinants for final treatment failure as for initial treatment failure. Additionally, having travelled abroad, contact with livestock, perineal carriage and having side-effects of the treatment were independently associated with final treatment failure. Household size, contact with livestock and MLST were left out of the multivariable analysis due to high multicollinearity with MRSA source, as certain sequences are predominantly found in specific sources. The multivariable analysis showed that the following determinants were associated with treatment failure: living in a refugee centre (OR_a_ 39.8, 95% CI [2.7–582.9]), age 0–17 (OR_a_ 5.0, 95% CI [1.1–22.5]), and having one or more comorbidities (OR_a_ 4.7, 95% CI [1.3–17.9]). We found that 92.9% of patients with a refugee status had final treatment failure, compared to 16.1% of non-refugees. In only one refugee, treatment was ultimately successful. Seven patients with a refugee status with initial treatment success were lost to follow-up and could not be included in this analysis.

**Table 3:** Variables associated with final treatment failure

	Total (*n* = 107)	Final treatment success (*n* = 79)	Final treatment failure (*n* = 28)	Crude Odds Ratio [95% CI]	*P*-value uni-variable	Adjusted Odds Ratio [95% CI]	*P*-value multi-variable
*Age, median*	33	39	15				
*Age groups, n (%)*							
0–17	30	13 (43.3)	17 (56.7)	6.41 [2.38–17.28]	< 0.001	4.97 [1.10–22.48]	0.04
18–64	61	51 (83.6)	10 (16.4)	Reference		Reference	
65 +	16	15 (93.8)	1 (6.3)	0.33 [0.04–2.43]	0.30	0.88 [0.08–10.20]	0.09
*Gender, n (%)*							
Male Female	57 50	40 (70.2) 39 (78.0)	17 (29.8) 11 (22.0)	Reference 1.50 [0.57–4.05]	0.39		
*Healthcare worker (HCW), n (%)*							
No Yes	94 13	66 (70.2) 13 (100.0)	28 (29.8) 0 (0.0)	Reference 0.00 [not applicable]			
*Contact with livestock, n (%)*							
No Yes/in the past	88 19	62 (70.5) 17 (89.5)	26 (29.5) 2 (10.5)	Reference 0.27 [0.03–1.29]	0.09		
*Pets*							
No Yes	76 31	56 (73.7) 23 (74.2)	20 (26.3) 8 (25.8)	Reference 0.94 [0.31–2.64]	1		
*MRSA source, n (%)*							
Unknown ^a^	20	17 (85.0)	3 (15.0)	Reference		Reference	
Hospital-acquired	26	21 (80.8)	5 (19.2)	1.27 [0.25–5.76]	0.77	1.24 [0.15–10.51]	0.84
Livestock-associated	18	16 (88.9)	2 (11.1)	0.67 [0.09–4.28]	0.68	1.76 [0.20–15.68]	0.61
Community-acquired	29	24 (82.8)	5 (17.2)	1.16 [0.24–5.49]	0.85	1.21 [0.19–7.85]	0.84
Living in refugee centre	14	1 (7.1)	13 (92.9)	69.33 [6.43–748.07]	< 0.001	39.81 [2.72–582.86]	< 0.01
*Comorbidity, n (%) *^*b*^							
No Yes	71 36	57 (80.3) 22 (61.1)	14 (19.7) 14 (38.9)	Reference 2.48 [0.93– 6.67]	0.06	Reference 4.72 [1.25–17.92]	0.02
Chronic pulmonary disease	9	5 (55.6)	4 (44.4)	2.38 [0.44–12.07]	0.24		
Skin disease	11	6 (54.5)	5 (45.5)	2.55 [0.56–11.09]	0.16		
Renal disease ^c^	6	4 (66.4)	2 (33.3)	1.40 [0.12–10.44]	0.66		
Devices ^d^	17	10 (58.8)	7 (41.2)	2.21 [0.63–7.42]	0.15		
*AB use in the past 3 months, n (%) *^*e*^							
No Yes	85 22	64 (75.3) 15 (68.2)	21 (24.7) 7 (31.8)	Reference 1.49 [0.45–4.63]	0.58		
*Hospital admission in the Netherlands in the past year, n (%) *^*e*^							
No Yes	84 23	63 (75.0) 16 (69.6)	21 (25.0) 7 (30.4)	Reference 1.27 [0.39–3.84]	0.79		
*Skin lesions, n (%)*							
No Yes	67 40	48 (71.6) 31 (77.5)	19 (28.4) 9 (22.5)	Reference 0.879 [0.27–2.12]	0.65		
*Travel abroad in the past year, n (%) *^*e,*^^f^							
No	72	59 (81.9)	13 (18.1)	Reference		Reference	
Yes	35	20 (57.1)	15 (42.9)	3.48 [1.29–9.60]	> 0.01	3.20 [0.70–14.54]	0.13
*Type of MRSA carriage, n (%)*							
Uncomplicated carriage Complicated carriage Infection & Complicated carriage	7 74 26	4 (57.1) 56 (75.7) 19 (73.1)	3 (42.9) 18 (24.3) 7 (26.9)	Reference 0.43 [0.09–2.10] 0.55 [0.10–3.12]	0.30 0.50		
*Location of MRSA carriage, n (%)*							
Nasal carriage	61	45 (73.8)	16 (26.2)	1.00 [0.39–2.66]	1	0.50 [0.13–1.93]	0.32
Non-nasal carriage	46	34 (73.9)	12 (26.1)	1.00 [0.38–2.61]	1		
Throat carriage	68	51 (75.0)	17 (25.0)	0.79 [0.30–2.16]	0.65		
Perineal carriage	45	38 (84.4)	7 (15.6)	0.36 [0.12–1.02]	0.04		
Other sites	31	23 (74.2)	8 (25.8)	1.07 [0.35–3.02]	1		
MLST							
ST398	28	24 (85.7)	4 (14.3)	Not applicable			
ST1	5	4 (80.0)	1 (20.0)				
ST8 and ST281 ^g^	8	4 (50.0)	4 (50.0)				
ST22	11	4 (36.4)	7 (63.6)				
ST30	12	10 (83.3)	2 (16.7)				
ST1933	3	1 (33.3)	2 (25.0)				
Other or Unknown	40	32 (80.0)	8 (20.0)				
*PVL positivity, n (%)*							
No Yes	86 21	64 (74.4) 15 (71.4)	22 (25.6) 6 (28.6)	Reference 1.34 [0.37–4.36]	0.58		
*Side-effects of treatment*							
No Yes	87 20	61 (70.1) 18 (90.0)	26 (29.9) 2 (10.0)	Reference 0.25 [0.03–1.19]	0.09	0.36 [0.04–3.06]	0.35
*Household tested*							
Yes No ^h^	97 10	74 (76.3) 5 (50.0)	23 (23.7) 5 (50.0)	Reference 3.13 [0.83–11.78]	0.09	Reference 5.41 [0.70–41.86]	0.11
*Size household*							
1–2 3–5 > 5 and unknown^i^	39 50 18	32 (82.1) 39 (78.0) 8 (44.4)	7 (17.9) 11 (22.0) 10 (55.6)	Reference 1.21 [0.42–3.49] 5.36 [1.55–18.54]	0.73 < 0.01		

^a^Known risk factors are: previous MRSA carriage, contact with livestock, contact with MRSA positive person, hospital admission outside the Netherlands

^b^The comorbidities mentioned below were merged together to get a larger sample size

^c^Solid organ transplant (SOT) or dialysis

^d^Tubing, catheters, probes, cannulas etc.

^e^Measured counting from first visit infectious diseases consult

^f^Travelling either to Asia, Africa, Central America or South America

^g^Strains associated with refugees[15]

^h^When one or more household members were not tested for MRSA, the index patient was categorized as ‘No’

^i^Unknown cases included refugees who fled alone, but had shared facilities at the refugee centre

### Children

In total, 35 patients were 0–17 years old (27.6%) and were treated at the paediatric clinic. Patient characteristics of this subgroup can be found in Tables 4, 5, and 6. Whereas 27/92 (29.3%) of the adults had relevant comorbidities, 12/35 (34.3%) of the children had comorbidities. All four children who were adopted had a cleft lip or palate. MRSA from these children were categorized as hospital-acquired MRSA as the children were all partially treated for their cleft lip or palate in their country of origin. In 42.9% of the patients, the source of MRSA was identified as caused by living in a refugee centre. Of the total number of refugees, 71.4% were children. The initial treatment success rate was 42.9%. The highest treatment success rates were seen in children with perineal carriage (70% initial treatment success), compared to nasal carriage (50%), and throat carriage (37%).

**Table 4:** Patient characteristics of adults and children

	Total (*n* = 127)	Children (*n* = 35)	Adults (*n* = 92)
Age, median	33	9	46
*Gender, n (%)*			
Male Female	66 61	14 (21.2) 21 (34.4)	52 (78.8) 40 (65.6)
*Pets*			
No Yes	92 35	25 (27.2) 10 (28.6)	67 (72.8) 25 (71.4)
*MRSA source, n (%)*			
Unknown ^a^	22	3 (13.6)	19 (86.4)
Hospital-acquired	30	5 (13.7)	25 (83.3)
Livestock-associated	21	2 (9.5)	19 (90.5)
Community-acquired	33	10 (30.3)	23 (69.7)
Living in refugee centre	21	15 (71.4)	6 (28.6)
*Comorbidity, n (%) *^*b*^			
No Yes	88 39	23 (26.1) 12 (30.8)	65 (73.9) 27 (69.2)
Chronic pulmonary disease	10	2 (20.0)	8 (80.0)
Skin disease	12	4 (33.3)	8 (66.7)
Renal disease ^c^	7	4 (57.1)	3 (42.9)
Devices ^d^	18	6 (33.3)	12 (66.7)
*AB use in the past 3 months, n (%) *^*e*^			
No Yes	99 28	32 (32.3) 3 (10.7)	67 (67.7) 25 (89.3)
*Hospital admission in the Netherlands in the past year, n (%) *^*e*^			
No Yes	101 26	30 (29.7) 5 (19.2)	71 (70.3) 21 (80.8)
*Skin lesions, n (%)*			
No Yes	84 43	28 (33.3) 7 (16.3)	56 (66.7) 36 (83.7)
*Travel abroad in the past year, n (%) *^*e, f*^			
No Yes	86 41	20 (23.3) 11 (36.6)	66 (76.7) 26 (63.4)
*Type of MRSA carriage, n (%)*			
Uncomplicated carriage	7	4 (57.1)	3 (42.9)
Complicated carriage	91	29 (31.9)	62 (68.1)
Infection & Complicated carriage	29	2 (6.9)	27 (93.1)
*Location of MRSA carriage, n (%)*			
Nasal carriage	70	18 (25.7)	52 (74.3)
Non-nasal carriage	57	17 (29.8)	40 (70.2)
Throat carriage	83	27 (32.5)	56 (67.5)
Perineal carriage	52	10 (19.2)	42 (80.8)
Other sites	34	5 (14.7)	29 (85.3)
*PVL positivity, n (%)*			
No Yes	104 23	30 (28.8) 5 (21.7)	74 (71.2) 18 (78.3)

^a^ Known risk factors are: previous MRSA carriage, contact with livestock, contact with MRSA positive person, hospital admission outside the Netherlands

^b^ The comorbidities mentioned below were merged together to get a larger sample size

^c^ Solid organ transplant (SOT) or dialysis

^d^ Tubing, catheters, probes, cannulas etc.

^e^ Measured counting from first visit infectious diseases consult

^f^ Travelling either to Asia, Africa, Central America or South America

**Table 5:** Variables associated with initial treatment failure in children

	Total (*n* = 35)	Initial treatment success (*n* = 15)	Initial treatment failure (*n* = 20)	*P*-value univariable
Age, median	9	7	10.5	Not applicable
*Gender, n (%)*				
Male Female	14 21	6 (42.9) 9 (42.9)	8 (57.1) 12 (57.1)	1
*Adopted and cleft lip/palate, n (%)*				
No Yes	31 4	12 (38.7) 3 (75.0)	19 (61.3) 1 (25.0)	Not applicable
*Pets*				
No Yes	25 10	12 (48.0) 3 (30.0)	13 (52.0) 7 (70.0)	0.46
*MRSA source, n (%)*				
Unknown ^a^	3	2 (66.7)	1 (33.3)	Not applicable
Hospital-acquired	5	3 (60.0)	2 (40.0)	
Livestock-associated	2	2 (100.0)	0 (0.0)	
Community-acquired	10	4 (40.0)	6 (60.0)	
Living in refugee centre	15	4 (26.7)	11 (73.3)	
*Comorbidity, n (%) *^*b*^				
No Yes	23 12	11 (47.8) 4 (33.3)	12 (52.2) 8 (66.7)	0.49
Chronic pulmonary disease	2	1 (50.0)	1 (50.0)	Not applicable
Skin disease	4	2 (50.0)	2 (50.0)	Not applicable
Renal disease ^c^	4	1 (25.0)	3 (75.0)	Not applicable
Devices ^d^	6	1 (16.7)	5 (83.3)	Not applicable
*AB use in the past 3 months, n (%) *^*e*^				
No Yes	32 3	14 (43.8) 1 (33.3)	18 (56.3) 2 (66.7)	Not applicable
*Hospital admission in the Netherlands in the past year, n (%) *^*e*^				
No Yes	30 5	13 (43.3) 2 (40.0)	17 (56.7) 3 (60.0)	1
*Skin lesions, n (%)*				
No Yes	28 7	10 (35.7) 5 (71.4)	18 (64.3) 2 (28.6)	0.11
*Travel abroad in the past year, n (%) *^*e, f*^				
No Yes	20 11	9 (45.0) 6 (40.0)	11 (55.0) 9 (60.0)	1
*Type of MRSA carriage, n (%)*				
Uncomplicated carriage	4	2 (50.0)	2 (50.0)	Not applicable
Complicated carriage	29	12 (41.4)	17 (58.6)	
Infection & Complicated carriage	2	1 (50.0)	1 (50.0)	
*Location of MRSA carriage, n (%)*				
Nasal carriage	18	9 (50.0)	9 (50.0)	0.50
Non-nasal carriage	17	6 (35.3)	11 (64.7)	0.50
Throat carriage	27	10 (37.0)	17 (63.0)	0.06
Perineal carriage	10	7 (70.0)	3 (30.0)	0.25
Other sites	5	1 (20.0)	4 (80.0)	0.67
*PVL positivity, n (%)*				
No Yes	30 5	14 (46.7) 1 (20.0)	16 (53.3) 4 (80.0)	0.29

^a^ Known risk factors are: previous MRSA carriage, contact with livestock, contact with MRSA positive person, hospital admission outside the Netherlands

^b^ The comorbidities mentioned below were merged together to get a larger sample size

^c^ Solid organ transplant (SOT) or dialysis

^d^ Tubing, catheters, probes, cannulas etc.

^e^ Measured counting from first visit infectious diseases consult

^f^ Travelling either to Asia, Africa, Central America or South America

**Table 6:** Variables associated with final treatment failure in children

	Total (*n* = 30)	Final treatment success (*n* = 13)	Final treatment failure (*n* = 17)	*P*-value univariable
Age, median	8.5	6	10	Not applicable
*Gender, n (%)*				
Male Female	14 16	6 (42.9) 7 (43.8)	8 (57.1) 9 (56.3)	1
*Adopted and cleft lip/palate, n (%)*				
No	26	10 (38.5)	16 (61.5)	
Yes	4	3 (75.0)	1 (25.0)	0.29
*Pets*				
No Yes	20 10	8 (40.0) 5 (50.0)	12 (60.0) 5 (50.0)	0.71
*MRSA source, n (%)*				
Unknown ^a^	3 5	2 (66.7) 3 (60.0)	1 (33.3) 2 (40.0)	Not applicable
Hospital-acquired	2	2 (100.0)	0 (0.0)	
Livestock-associated	9	5 (55.6)	4 (44.4)	
Community-acquired Living in refugee centre	11	1 (9.1)	10 (90.9)	
*Comorbidity, n (%) *^*b*^				
No Yes	18 12	8 (44.4) 5 (41.7)	10 (55.6) 7 (58.3)	1
Chronic pulmonary disease Skin disease Renal disease ^c^ Devices ^d^	2 4 4 6	1 (50.0) 1 (25.0) 3 (75.0) 2 (33.3)	1 (50.0) 3 (75.0) 1 (25.0) 7 (66.7)	
*AB use in the past 3 months, n (%) *^*e*^				
No Yes	27 3	12 (44.4) 1 (33.3)	15 (55.6) 2 (66.7)	1
*Hospital admission in the Netherlands in the past year, n (%) *^*e*^				
No Yes	25 5	11 (44.0) 2 (40.0)	14 (56.0) 3 (60.0)	1
*Skin lesions, n (%)*				
No Yes	23 7	8 (34.8) 5 (71.4)	15 (65.2) 2 (28.6)	0.19
*Travel abroad in the past year, n (%) *^*e, f*^				
No Yes	18 12	10 (55.6) 3 (25.0)	8 (44.4) 9 (75.0)	0.14
*Type of MRSA carriage, n (%)*				
Uncomplicated carriage	4	2 (50.0)	2 (50.0)	Not applicable
Complicated carriage	24	11 (45.8)	13 (54.2)	
Infection & Complicated carriage	2	0	2 (100.0)	
*Location of MRSA carriage, n (%)*				
Nasal carriage	18	8 (44.4)	10 (55.6)	1
Non-nasal carriage	12	5 (41.7)	7 (58.3)	1
Throat carriage	23	9 (39.1)	14 (60.9)	0.67
Perineal carriage	9	5 (55.6)	4 (44.4)	0.44
Other sites	5	0 (0.0)	5 (100.0)	0.05
*PVL positivity, n (%)*				
No Yes	25 5	12 (48.0) 1 (20.0)	13 (52.0) 4 (80.0)	0.35

^a^Known risk factors are: previous MRSA carriage, contact with livestock, contact with MRSA positive person, hospital admission outside the Netherlands

^b^ The comorbidities mentioned below were merged together to get a larger sample size

^c^ Solid organ transplant (SOT) or dialysis

^d^ Tubing, catheters, probes, cannulas etc.

^e^ Measured counting from first visit infectious diseases consult

^f^ Travelling either to Asia, Africa, Central America or South America

The final treatment outcome was successful in 43.3% (13/30) of the patients. In the complicated carriers, the final treatment outcome rate was successful in 42.3% (11/26). The lowest final treatment success rates were seen in children living in a refugee centre (1/11; 9.1%) compared to other sources of infection (56–100%) success rates) and children with carriage in ‘other sites’ than nose, throat, and perineum. These cultures from ‘other sites’ were obtained from a wound, sputum or eyes.

## Discussion

Up until now, there has been limited research on the risk factors of failure of MRSA decolonization treatment. The previous Dutch study was published in 2011 and had an initial treatment success rate of 56% among complicated carriers [2]. Success rates have not been evaluated in nearly 10 years while there have been important changes in guidelines, demographics, and MRSA epidemiology. Compared to this previous research [2], the treatment success rate of the Radboudumc [9] is higher. This study showed a final treatment success rate of MRSA decolonization therapy of 75% for complicated carriers. There was a limited number of uncomplicated carriers, as they are normally treated by their GP and not referred to an MRSA outpatient clinic [7]. In our study, all uncomplicated carriers were household members of index patients with a complicated carriage and were therefore treated simultaneously at the clinic. When looking at final treatment failure, our results showed that patients aged 0–17 years old, patients with relevant comorbidities, and patients living in a refugee centre had a significantly higher risk of treatment failure. The same risk factors were identified for initial treatment failure, with an addition of patients whose household members were not tested for MRSA.

The previous Dutch study showed that having chronic pulmonary disease, activities of daily living (ADL) dependency and the presence of devices, were strong predictors of treatment failure [2]. Another important finding was that treatment failure is associated with not testing and treating household members [2]. Similar results were found in a study conducted, in which it was observed that devices, relevant comorbidities and ADL dependency are independently associated with MRSA treatment failure [10]. In our study, the majority of household members were tested for MRSA. Since previous research only used three consecutive negative culture sets to determine treatment failure, the results from our initial treatment failure were used for comparison. In the multivariable analysis of our data, the probability of treatment failure is higher when one or more household members were not tested, thus confirming the results of previous research. This was not seen in the multivariable analysis of final treatment failure. This could possibly be explained by testing household members in latter decolonization attempts that were initially not tested. Our study also confirmed having relevant comorbidities were associated with treatment failure. Therefore, our results support previous research and the updated Dutch national protocol.

Other factors that have been described as risk factors for MRSA eradication treatment failure are recent antibiotic use, presence of a skin wound, previous hospitalization, and older age [11]. Although these variables have been tested in our study, we did not find an association with failed MRSA decolonization. This could be partially explained by the older age of the study population compared to our study, as children were not included in this study [11]. In our study, age 0–17 years was associated with treatment failure (OR_a_ 4.1, 95% CI [1.2–14.5]), while older age was not. This can be explained by the high percentage and severity of the underlying comorbidity in the children included in our study. Treatment compliance could also be of concern. Additionally, part of the children were refugees, which was also shown to be an important risk factor for treatment failure. Because of this, these children were not representative of the general population. To our knowledge, this is the first study that has also looked specifically at MRSA decolonization treatment in children. Our findings do not imply that decolonization treatment in children should be discouraged.

As stated previously, research has shown that ADL dependency may be associated with treatment failure [2, 10]. Another study also suggested that tonsillectomy might be able to improve treatment success in persistent MRSA carriers [12]. Unfortunately, due to the lack of systematic registration of these variables in medical records, this could not be analysed in our study. Additional information on antibiotic resistance and side effects of the treatment could provide further insight as well. Therefore, a prospective study with preferably a larger sample size is still favourable to fully assess the impact of all risk factors of interest.

A remarkable difference between our study and the previous Dutch study is that the Netherlands has been receiving more refugees since 2015 [13, 14]. Refugees are often from countries with a higher prevalence of resistant micro-organisms [15–17]. To our knowledge, this is the first study on MRSA decolonization that takes refugee status into account as potential risk factor. Refugee status was associated with treatment failure. Many refugees were lost to follow-up as MRSA recurrence was often seen in their household members, which led to the termination of collecting more cultures, or because of transfer to another refugee centre. Our local public health and infectious diseases specialists observed that successful decolonization may be harder to achieve in people living in a refugee centre, which was confirmed by this study. Refugees are often not treated for MRSA due to the continuous exposure to other people with MRSA. They often only receive treatment if there is a high medical urgency such as a planned surgery or a medical condition. Additionally, many refugees have large households and several households usually share facilities in a refugee centre. This makes it harder and less feasible to test all household members, which is a risk factor for treatment failure. People living in a refugee centre often face less optimal hygienic living conditions. According to guidelines, it is recommended that patients change their clothing, bedding and towels regularly when being treated for MRSA [7]. This might not be feasible for people living in a refugee centre as there are often restrictions when using washing machines. With the current situation in Ukraine, many EU countries, including the Netherlands, will be welcoming Ukrainian refugees. Data have shown that high rates of antimicrobial resistance, including MRSA, are reported in Ukraine [18, 19]. Therefore, these findings may also have an important implication for treating Ukrainian refugees.

A practical challenge is that many refugees in the Netherlands have financial restrictions. Although antibiotics are covered by medical insurance, the recommended antiseptics are not. These costs are often a barrier that cannot be overcome and because of which treatment is eventually postponed until these patients have better living and financial conditions. This highlights the importance of involving the refugee centres and regional public health services to improve the conditions of refugees to achieve higher success rates. Finally, antibiotic policy in the Netherlands often differs from other countries. There is a likelihood that refugees might be colonized with MRSA types that are harder to treat.

This was an observational retrospective cohort study, that had some limitations. Due to being a single-centre study the sample size was relatively small. Furthermore, people who visit the MRSA outpatient clinic or the paediatric clinic often already have several risk factors or comorbidities, which can influence treatment outcome negatively. This may have led to a higher prevalence of certain risk factors. The overall treatment success rates that were found in our study may therefore be lower compared to MRSA treatment success rates in primary care or regional hospitals. An additional uncontrolled factor is that not everyone who visits the MRSA outpatient clinic is treated. In patients with a continuous exposure to MRSA no decolonization attempt was started as it was considered highly likely that treatment would fail. In this group, only in patients with high medical urgency, load reduction or decolonization was attempted. High urgency also indicates that the patient may have underlying problems.

It was not possible to assess all patient data of HCWs who were treated for MRSA due to privacy reasons. As a result, not all relevant HCWs could be included in this study. This could have contributed to a more biased selection. For patients who were referred to the MRSA outpatient clinic, the MRSA genotyping sequence was not always available. Likewise, cultures of re-colonized patients were often not re-typed to assess whether the patient was colonized with a new strain. We assumed that these patients had a recurrence rather than a reinfection, as MRSA prevalence in the Netherlands is only 1.2% [3]. Information regarding MLST types was however limited and could not be used for the multivariable regression analysis.

## Conclusion

In conclusion, current treatment success of MRSA carriage based on the SWAB guideline is good. Since previous research has shown that transmission occurs in about half of the cases from an index person to household members, current success rates are likely due to the renewed policy where household members are tested and treated at the same time [20]. Household members should still be tested and treated at the same time as the index patient. One of the more significant findings to emerge from this study is that patients with a refugee status and children with important comorbidity have a higher probability of treatment failure. For this reason, it might be useful to revise decolonization strategies for these groups and to refer these patients to specialized outpatient clinics. For people living in refugee centres, it seems to be important to also involve other parties, such as the refugee centres and regional public health services, to address these difficulties.

## Data Availability

The datasets generated and analysed during the current study are not publicly available due to privacy reasons but are available from the corresponding author on reasonable request.

## Abbreviations

ADL: Activities of daily living
HCW: Healthcare worker
LA-MRSA: Livestock associated MRSA
MRSA: Methicilin-resistant *Staphylococcus aureus*
PVL: Panton-Valentine Leukocidin
S&D: Search and destroy
SOT: Solid organ transplant
SWAB: Stichting werkgroep antibioticabeleid
VIF: Variance Inflation Factor
WIP: Werkgroep Infectie Preventie
